# Perception and attitudinal factors contributing to periodic deworming of preschool children in an urban slum, Nigeria

**DOI:** 10.1186/s12889-020-09958-x

**Published:** 2020-12-01

**Authors:** Paul Eze, Ujunwa Justina Agu, Chioma Lynda Aniebo, Sergius Alex Agu, Lucky Osaheni Lawani

**Affiliations:** 1 Department of Health Policy and Administration, Penn State University, University Park, PA 16802 USA; 2 Department of Paediatrics, Enugu State University Teaching Hospital, Parklane, Enugu, Nigeria; 3 Department of Paediatrics, University of Nigeria Teaching Hospital, Ituku-Ozalla, Enugu, Nigeria; 4 Institute of Health Policy, Management and Evaluation, University of Toronto, Toronto, ON M5T 3M6 Canada

**Keywords:** Soil-transmitted helminths, Preschool-aged children, Deworming, Periodic chemotherapy, Mixed-methods, Urban slum, Nigeria

## Abstract

**Background:**

Over 20 million preschool-age children (PSAC) in Nigeria require periodic chemotherapy (PC) for soil-transmitted helminth (STH) infections. Persistently low coverage for this age group threatens the World Health Organization (WHO) 2030 target for eliminating STH infections. Current strategies for targeting PSAC have been largely ineffective. Hence, PSAC are mostly dewormed by their parents/caregivers. However, little is known of the perception and attitude of parents/caregivers of PSAC to deworming in this setting.

**Methods:**

A mixed methods design, combining a community-based interviewer-administered questionnaire-survey (*n* = 433) and focus group discussions (FGD) (*n* = 43) was used to assess the perceptions and attitudes of mothers to periodic deworming of preschool children aged 2–5 years in Abakpa-Nike, Enugu, Nigeria.

**Results:**

Coverage of periodic deworming in PSAC is 42% (95% CI: 37.3–46.8%). There is significant difference in the specific knowledge of transmission of STH (AOR = 0.62, 95% CI: 0.48–0.81, *p* = 0.000), complication of STH infections (AOR = 0.77, 95% CI: 0.61–0.98, *p* = 0.034), accurate knowledge of deworming frequency (AOR = 0.41, 95% CI: 0.18–0.90, *p* = 0.026), and knowledge of PC drug, mebendazole (AOR = 0.28, 95% CI: 0.09–0.90, *p* = 0.031), and pyrantel (AOR = 8.03, 95% CI: 2.22–29.03, *p* = 0.001) between mothers who periodically deworm their PSAC and those who do not. There is no significant difference in specific knowledge of the symptoms of STH infections (AOR = 0.76, 95% CI: 0.57–1.02, *p* = 0.069) and PC drug, Albendazole (AOR = 1.00, 95% CI: 0.46–2.11, *p* = 0.972). FGD revealed misconceptions that are rooted in stark ignorance of the disease. Overall attitude to deworming is positive and favourable.

**Conclusions:**

Poor coverage of periodic deworming for STH infections in PSAC in this setting are primarily driven by poor specific knowledge of the risks and burden of the infection. Focused health education on the burden and transmission of STH infections could complement existing strategies to improve periodic deworming of PSAC in this setting.

**Supplementary Information:**

The online version contains supplementary material available at 10.1186/s12889-020-09958-x.

## Background

Playing in the sand and getting dirty is part of childhood, but these innocent childhood fun activities put millions of children in developing countries at risk of contracting soil-transmitted helminths (STH) – intestinal worms dwelling in humans that are transmitted through contaminated soil [1, 2]. One of the three main STH infections – Roundworm (*Ascaris lumbricoides*), Whipworm (*Trichuris trichiura*), and Hookworms (*Ancylostoma duodenale* and *Necator americanus*), commonly parasitize the gastrointestinal tract of children in developing countries, and in many cases all three worms are present [1]. STHs are the most prevalent (> 1.6 billion people infected) and most burdensome (global burden of 3.486 million disability-adjusted life years) neglected tropical diseases [3, 4]. Though STH infections are not a leading cause of death, they are an extremely important cause of childhood physical and intellectual retardation and have profound effects on future school attendance, academic performance, and economic productivity [1, 4].

Even light infection with STH can cause pain, discomfort, and illness [5]. Moderate to heavy STH infection in PSAC can lead to mal-absorption, vitamin A deficiency, iron deficiency anemia, and malnutrition [5–7]. Chronic STH infections can dramatically affect physical and mental development in children [1, 2]. These worms mostly affect children living in extreme poverty, particularly those living in rural communities or urban slums that lack adequate water, sanitation, and hygiene (WASH) [4]. Due to childhood behaviors, preschool-aged children (PSAC) – defined as children between the ages of 2 to 5 years, are particularly prone to STH infections [8]. Playing in the mud, sucking their fingernails, and eating soil/sand easily exposes these children to the eggs and larvae of these parasites. Additionally, PSACs are less likely to have developed habits of washing hands before a meal nor toilet hygiene [6, 9]. STH infection risk in this age group is heightened when this is coupled with low socioeconomic status, low educational status of parents, and poor WASH facilities. STH infections indeed represent an important public health problem, especially for children in low- and middle-income countries (LMIC) [10].

Periodic deworming with an inexpensive, safe, effective, single-tablet treatment - albendazole (400 mg) and mebendazole (500 mg), drastically relieves children infected with STH of the morbidities of the infection [11], and sometimes reverses the growth and physical fitness deficits caused by chronic STH infections [2]. Due to the sensitive and critical developmental formations that occur before the age of five, periodic deworming is, without a doubt, important in PSACs in endemic areas [8, 12, 13]. This is especially important in Nigeria where the prevalence of STH in this age group is high at 58.3% [14]. The World Health Organization (WHO) in 2018 estimates that 20,046,813 PSACs in Nigeria require treatment for STH infections [15], and given the high prevalence (> 50%) of STH infection in Nigeria, WHO recommends that these children be periodically dewormed every 4–6 months [16, 17].

However, school-based mass drug administration (MDA) programs do not cover PSAC and child health programs or the healthcare system are ineffective in reaching this age group [8, 12, 16, 18–20]. Hence, PSACs in our setting are mostly self-dewormed by the parents/caregivers. However, little is known of the perception and attitude of parents/caregivers of PSAC to deworming for STH infections in this setting. Our study aims to fill this gap. We hypothesize that mothers with higher awareness of STH, transmission, symptoms, and complications of STH infections will have a better perception of deworming and are more likely to periodically deworm their PSACs. Our study findings may be useful in improving existing interventions for STH elimination in Enugu State and in other similar settings.

## Methods

### Study design and period

This is a mixed methods study involving both quantitative and qualitative study methods. The study was conducted in January and February 2020 among mothers of children aged 2–5 years in Abakpa-Nike, Enugu East Local Government Area (LGA), Enugu State, Nigeria.

The Strengthening the Reporting of Observational Studies in Epidemiology (STROBE) guideline was used to ensure appropriate reporting of our study’s design, conduct and findings [21], while the FGD sessions are reported according to the consolidated criteria for reporting qualitative research framework [22, 23].

### Study setting

Abakpa-Nike – the urban extension of the Nike community, is a densely populated slum in Enugu East LGA. The 2016 projected population of Enugu East LGA is 374,100, with most of the population living in Abakpa-Nike [24]. Abakpa-Nike is composed of five neighborhoods: Ugboghe, Ogwuago, Ugbene I, Ugbene II, and Ugboezeji [25]. The main religion in the community is Christianity and the major occupations are farming, trading, civil service, artisans of different trades, e.g. carpentry, mechanics, electricians, and daily-paid labour [26]. Abakpa-Nike has inadequate infrastructures for safe drinking water, sanitation, and hygiene [27, 28].

### Quantitative study / community-based cross-sectional survey

#### Study participants

All consenting mothers of PSAC aged 2–5 years who were available during the survey period were eligible to participate.

#### Sample size

The sample size for the survey was calculated based on a single proportion formula by assuming 50% coverage to obtain the maximum sample size [29]. Based on a design effect (DEFF) of 1.05 obtained by pretesting the questionnaire, significance of 5.0%, precision (margin of error) of 5.0%, and an inflation of 5.0% (to account for non-response), the estimated minimum sample was 426. This was then increased to 440 to boost the power of the study.

#### Sampling procedure

Multistage sampling was used to identify the mothers to be interviewed. Each neighborhood in Abakpa-Nike was divided into four clusters. Twenty-two households were identified from each cluster using a 1 in 5 systematic sampling method after an initial random sampling to interview the fifth household. Mothers in the selected households who met the inclusion criteria were recruited. The inclusion criteria were every available consenting mother with a child aged 2 to 5 years. Exclusion criteria were mothers who did not live for 1 year in the study area and mothers who refused consent. Where the mother had more than one child in this age group, we asked her to give answers based on the youngest child in that age bracket.

#### Data collection

Questionnaires were paper-based and administered in either English or Igbo languages depending on the language preference of the mother. Questions were directed at the mothers and only her responses were collected. The questionnaires were interviewer-administered by five teams of paired female social workers (each pair had at least one mother) who had basic medical experience, suitable communication skills, competent in both English and Igbo languages, and were trained for a full day prior to data collection. Study questionnaires were piloted on 25 mothers to ensure internal validity. The validity and reliability of the instrument were ascertained prior to final administration. The Cronbach alpha correlation was 0.80. Data collection was directly supervised by three of the authors with technical support from the other authors.

#### Study variables

*Demographic information*: This information included mothers’ age in years, marital status, educational level, and occupation. Other demographic information collected were the fathers’ occupation, household income and religion, sex of the index child, and the number of children aged 2–5 years in the family.

*Mothers’ knowledge of STH*: we first assessed mothers’ general familiarity with STH and the source of their knowledge of STH. This was followed by 20 questions on the knowledge of the different STH, modes of transmission of STH infection, symptoms of STH infection, and main complications of STH infections in PSAC to assess mothers’ knowledge of STH infections. The response options were “Yes” or “No”. A response of Yes was scored 1 and No was scored 0. Total scores ranged from 0 to 20.

*Mothers’ knowledge of deworming*: we assessed mothers’ general familiarity with deworming for STH infections in preschool children, how they got to know about periodic deworming, knowledge of common drugs for deworming for STH (albendazole, mebendazole, and pyrantel), and frequency of periodic deworming.

*Mothers’ attitude to periodic deworming*: we assessed mothers’ attitude to periodic deworming with a five-point Likert type scale – strongly disagree, disagree, indifferent, agree and strongly agree, and nine statements: “STH infection is a serious health infection in preschool children”, “Deworming is good for the health of preschool children”, “Deworming helps to prevent malnutrition in preschool children”, “Deworming helps to prevent growth retardation in preschool children”, “Deworming prevents shortage of blood in preschool children”, “Periodic deworming of preschool children is very difficult”, “Deworming of preschool children is quite expensive to do”, “Deworming is NOT necessary for the health of preschool children”, and “Deworming makes preschool children sick and should NOT be encouraged”.

*Mothers’ preventative measures against STH infection*: We then assessed preventative measures mothers take at home, source of household drinking water, toilet care for index child, care of fingernails, and frequency of wearing footwear. For mothers who have never dewormed their child, we asked for the reasons why they never dewormed their PSAC.

The outcome variable was periodic deworming of the index child aged 2–5 years in the last 12 months. Periodic deworming for this study was defined as the mother’s reported deworming of the index child at least twice in the past 12 months (January to December 2019) and the last deworming treatment was given within the 6 months prior to the interview (July to December 2019).

#### Data management and statistical analysis

Data were entered into Microsoft Excel® (Microsoft, Redmond, WA, USA), cleaned and transferred to IBM SPSS® version 25.0 (IBM, Armonk, NY, USA) for statistical analyses. 100% stacked bars were prepared using Microsoft Excel. Frequency and percentage were used to describe the data and Chi-square test was used to test for statistical significance. Scores for each knowledge domain were summed up to obtain an aggregate score for these knowledge domains. T-test was used to assess for statistical difference in the mean scores for knowledge scores. Mothers’ attitude to deworming were dichotomized; strongly disagree, disagree, and neither agree nor disagree responses were aggregated into one group while agree and strongly agree responses were aggregated into another group. Chi-Square analysis was used to assess the attitudinal difference between the two groups of mothers. Finally, multivariable logistics regression analyses were performed to assess for difference in the general familiarity with, specific knowledge of STH, transmission, symptoms, complications, and frequency of deworming while adjusting for potential confounders such as socio-demographic characteristics (mothers age, marital status, mothers educational status, mothers occupation, fathers occupation, religion, family monthly income, number of children < 5 years in the family, and sex of the index child), and source of information on STH infections and deworming. *P* < 0.05 was used to define statistical significance, and all tests were two-tailed.

### Qualitative study / focus group discussions (FGD)

#### Sample size and sampling strategy

Forty-three mothers participated in five FGD sessions, averaging at least 8 mothers per session. Mothers were purposely selected during the community-based survey, particularly inviting mothers who indicated that they had never dewormed their PSAC (four sessions) and mothers who do not periodically deworm their PSAC (one session).

#### Data collection instrument and data collection technique

Different semi-structured guides were developed and applied for focus group discussions. A moderator and a note-taker conducted each session using an FGD guide and a tape recorder. The FGD sessions were guided by themes with discourse analysis which evaluated mothers’ perception and attitude to deworming of their PSAC. FGD sessions were held after work hours at the Abakpa-Nike primary healthcare center, were led by two of the authors (both females) and the average duration of FGD sessions was about 40 min. The discussions were conducted in Igbo language as all the mothers preferred this language. Confidentiality was assured and mothers were encouraged to be frank with their contributions.

#### Data management and analysis

Audio records of the FGD were transcribed verbatim into Igbo by an external research assistant and was checked by two authors (UJA and CLA) who listened to the recording while checking the accuracy of the transcripts. A thematic analysis approach was adopted, and three authors (UJA, CLA, and PE) independently analyzed transcripts using comparative analysis from which themes were developed until data saturation [30]. Themes were discussed among the authors to clarify biases. The transcript of the audio records and the major findings from the analysis of these transcripts were cross-checked with a few FGD participants for corrections and validation. Selected texts were then translated into English when drafting this paper.

## Results

### Socio-demographic characteristics of mothers in the community-based survey

Out of 440 administered questionnaires, 433 were correctly filled and considered for analysis yielding a response rate of 98.4%. Over half of the mothers were aged 30 years and above (52%) and had post-secondary education (54.1%). Most of the mothers are married (94.9%) and Christian (94.0%). Two in three (67.4%) of the households earned less than N75,000 (about USD 200) per month. A little more than half (51.3%) of the index PSAC assessed were girls. About 1% of households have access to pipe-borne water. Other baseline sociodemographic characteristics of the mothers are shown in Table 1.

**Table 1:** Socio-demographic characteristics of study participants, *N* = 433

Baseline socio-demographic characteristics	Total (%)	Periodic deworming of Index child		***P***-value
		Yes (***n*** = 182)	No (***n*** = 251)	
**Mothers’ age**				
– < 20 years	7 (1.6%)	3 (1.6%)	4 (1.6%)	0.123
– 20–24 years	51 (11.8%)	26 (14.3%)	25 (10.0%)	
– 25–29 years	150 (34.6%)	52 (28.6%)	98 (39.0%)	
– ≥ 30 years	225 (52.0%)	101 (55.5%)	124 (49.4%)	
**Marital status**				
– Single	6 (1.4%)	1 (0.5%)	5 (2.0%)	0.210
– Married	411 (94.9%)	175 (96.2%)	236 (94.0%)	
– Widow/Divorced/Separated	16 (3.7%)	6 (3.3%)	10 (4.0%)	
**Mothers’ highest educational qualification**				
– No formal education	4 (0.9%)	1 (0.5%)	3 (1.2%)	0.718
– Primary	30 (6.9%)	11 (6.0%)	19 (7.6%)	
– Secondary	165 (38.1%)	67 (36.8%	98 (39.0%)	
– Post-secondary/Tertiary	234 (54.1%)	103 (56.7%)	131 (52.2%)	
**Mothers’ main occupation**				
– Stay-at-home Mom	83 (19.2%)	27 (14.8%)	56 (23.3%)	0.211
– Artisan/Lows skilled jobs	33 (7.6%)	13 (7.1%)	20 (8.0%)	
– Business/Trader	142 (32.8%)	61 (33.5%)	81 (32.3%)	
– Professionals/Civil servant	175 (40.4%)	81 (44.5%)	94 (37.5%)	
**Religion**				
– Christian	407 (94.0%)	174 (95.6%)	233 (92.8%)	0.262
– Muslim	15 (3.5%)	6 (3.3%)	9 (3.6%)	
– Traditionalist	11 (2.5%)	2 (1.1%)	9 (3.6%)	
**Fathers’ main occupation**				
– Absent/Late father	22 (5.1%)	7 (3.8%)	15 (6.0%)	0.351
– Artisan/Low skilled jobs	75 (17.3%)	34 (18.7%)	41 (16.3%)	
– Business/Trader	182 (42.0%)	70 (38.5%)	112 (44.6%)	
– Professionals/Civil Servant	154 (35.6%)	71 (39.0%)	83 (33.1%)	
**Sex of index child**				
– Female	222 (51.3%)	86 (47.3%)	136 (54.2%)	0.173
– Male	211 (48.7%)	96 (52.7%)	115 (45.8%)	
**Number of Children aged 2 to 5 years in the family**				
– 1 child	289 (66.7%)	110 (60.4%)	179 (71.3%)	0.056
– 2 children	102 (23.6%)	50 (27.5%)	52 (20.7%)	
– 3 children	42 (9.7%)	22 (12.1%)	20 (8.0%)	
**Total family monthly income** ^**a**^				
– < N75,000	292 (67.4%)	121 (66.5%)	171 (68.1%)	0.756
– ≥ N75,000	141 (32.6%)	61 (33.5%)	80 (31.9%)	
**Sources of household drinking water** ^**b**^				
– Sachet water	279 (64.4%)	110 (60.4%)	169 (67.3%)	0.155
– Tanker/Borehole	153 (35.3%)	70 (38.5%)	83 (33.1%)	0.263
– Pipe borne water	14 (0.9%)	7 (3.9%)	7 (2.8%)	0.589
– Rainwater	5 (1.2%)	3 (1.6%)	2 (0.8%)	0.654
– Stream water	4 (0.9%)	3 (1.6%)	1 (0.4%)	0.314

^a^1 USD = N360.00 on the Currency exchange market on 20 January 2020; www.oanda.com

^b^More than one water source can apply

### Socio-demographic characteristics of FGD participants

FGD participants were all married and lived with their husbands. Thirty-four had secondary education, seven had post-secondary education while two had primary education. Mothers in the FGD sessions were mostly housewives (32 mothers), while eight were traders and three civil servants. Nearly all of the mothers were Igbos, but one was Efik, though she was also fluent in Igbo. The age range was 23 to 38 years, and all the participants had lived in Abakpa-Nike for at least 2 years.

### Mothers’ perception of STH infections in PSAC

Most (98.6%) of the mothers acknowledged having previously heard of STH infections in PSAC and the most common source of knowledge was during hospital/health center visits (89.0%) – see Table 2. Other important sources were health talks on radio and TV (50.1%), school lectures (45.5%), and discussion with family and friends (42.2%). Only 6.8% of mothers reported knowing about STH on social media platforms (Facebook, WhatsApp, Twitter, and Instagram). There was no significant difference in the knowledge of the different STH between mothers who periodically dewormed their PSAC and those that did not periodically deworm their PSAC (*p* = 0.724). However, there was a statistically significant difference in the knowledge of the modes of transmission of STH infection (*p* = 0.001), knowledge of the different symptoms of STH infection in PSAC (*p* = 0.006), and knowledge of the common complications of STH infections in PSAC (*p* = 0.002) between mothers who periodically deworm their PSAC and those who do not.

**Table 2:** Knowledge of soil-transmitted helminthiasis among mothers in Abakpa-Nike, Enugu, Nigeria 2020. *N* = 433

Knowledge on different STH, transmission, symptoms, and complications of STH infection in preschool children	Total (%)	Periodic deworming of Index child		***P***-value
		Yes	No	
**Are you familiar with soil-transmitted helminthiasis?** ***N*** **= 433**				
– Yes	427 (98.6%)	182 (100.0%)	245 (97.6%)	0.042
– No	6 (1.4%)	0 (0.0%)	6 (2.4%)	
**How did you know about soil-transmitted helminthiasis?** ^**a**^ ***N*** **= 427**				
– During visits to a hospital or health center	380 (89.0%)	161 (88.5%)	219 (89.4%)	0.440
– Discussions with family and friends	180 (42.2%)	69 (37.9%)	111 (45.3%)	0.138
– Health talks on TV/Radio	214 (50.1%)	81 (44.5%)	133 (54.3%)	0.051
– Classes/lectures in school	197 (45.5%)	74 (40.7%)	123 (49.0%)	0.097
– Health talk in church/mosque	40 (9.4%)	14 (7.7%)	26 (10.6%)	0.320
– Social media (Facebook, WhatsApp, Twitter, Instagram)	29 (6.8%)	12 (6.6%)	17 (6.9%)	1.000
– Others (Women’ meeting = 10, In a travelling bus = 3)	13 (3.0%)	4 (2.2%)	9 (3.7%)	0.127
**Knowledge of the different STH in preschool children.** ***N*** **= 427**				
– Mean (± Std Dev)	1.93 (± 0.73)	1.94 (± 0.75)	1.91 (± 0.72)	0.724
**Knowledge of the modes of transmission of STH infection in preschool children.** ***N*** **= 427**				
– Mean (± Std Dev)	3.35 (± 1.19)	3.57 (± 0.98)	3.18 (± 1.29)	0.001
**Knowledge of the different symptoms of STH infection in preschool children.** ***N*** **= 427**				
– Mean (± Std Dev)	2.10 (± 0.90)	2.24 (± 0.76)	2.00 (± 0.98)	0.006
**Knowledge of the main complications of STH infections in preschool children.** ***N*** **= 427**				
– Mean (± Std Dev)	3.08 (± 1.20)	3.28 (± 1.04)	2.93 (± 1.29)	0.002
**Aggregate scores on different STH, transmission, symptoms, and complications of STH infection in preschool children,** ***N*** **= 427**				
– Mean (± Std Dev)	10.45 (± 2.82)	11.03 (± 2.24)	10.02 (± 3.12)	0.000

^a^More than one answer can apply

Three themes emerged from our FDG sessions: knowledge and misconceptions on STH transmission, awareness of the symptoms of STH infection, and knowledge of the complications of STH infections. Most mothers mentioned eating with contaminated fingers and drinking dirty water as sources of STH infections. However, there was a strongly- and widely-held misconception that excessive sugar consumption causes and exacerbates STH infections.

*“Mothers have to limit the amount of sugar they give their children. It is this sugar that causes these worms*.” Mrs. EN, FGD

*“… Ehen, you come to all these things that taste very sweet, there are some people that use it to train their children… there are parents that train their children with all these sweet and sugary things. Some parents don’t regard those things as harmful, meanwhile, they are the things that cause worm…”* Mrs. CU, FGD

*“… The mother is supposed to be giving the child something that is a little bitter, maybe sometimes the person should squeeze bitter leaf for the child to calm the problems of these worms.”* Mrs. NE, FGD

Other notable misconceptions were related to the transmission of STH infections. Study participants mentioned that STH infections could be transmitted by consuming poorly cooked beans (legumes), transmitted in breastmilk, and that children are born with the infection which worsens with consumption of sugar. Most FGD participants, however, were able to mention at least one symptom of STH infection. Although most mothers mentioned excessive weight loss and irritability (excessive crying), a few (six) will only suspect STH infections when they see the worms in stool. Mothers generally had poor knowledge of the complications of STH infections – only a couple of mothers recognized stunting and growth retardation as STH complications.

### Mothers’ perception of periodic deworming in PSAC

Coverage of periodic deworming in PSAC was 42% (95% CI: 37.3–46.8%). Most mothers (97.7%) acknowledged having previously heard of periodic deworming in PSAC – Table 3, and hospital visits were also the main source of awareness – 90.8%. Similarly, discussions with family and friends (61.9%), health talks on radio and TV (49.2%) and school lectures (41.6%) were other important sources of information on periodic deworming. Albendazole and pyrantel are more widely known anthelmintic drugs – about 58.2 and 57.7% respectively. More mothers who periodically deworm their PSAC know albendazole as a drug for periodic deworming than mothers who do not periodically deworm their PSAC (64.3% vs 53.5%, *p* = 0.029) while more mothers who do not periodically deworm their PSAC know about pyrantel than mothers who periodically deworm their PSAC (65.2% vs 47.8%, *p* = 0.001). Only about half of the mothers know mebendazole as a drug for periodic deworming of PSAC – about 51%.

**Table 3:** Mothers’ knowledge of deworming in preschool children in Abakpa-Nike, Enugu Nigeria 2020

Knowledge of periodic deworming	Total (%)	Periodic deworming of Index child		***P***-value
		Yes	No	
**Are you familiar with periodic deworming in preschool children?** ***N*** **= 433**				
– Yes	423 (97.7%)	182 (100.0%)	241 (96.0%)	0.006
– No	10 (2.3%)	0 (0.0%)	10 (4.0%)	
**How did you know about periodic deworming in preschool children?** ^**a**^ ***N*** **= 423**				
– During visits to the hospital or health center	384 (90.8%)	162 (89.0%)	222 (92.1%)	0.310
– Discussions with Family and friends	262 (61.9%)	70 (38.5%)	101 (41.9%)	0.485
– Health talks on TV/Radio	208 (49.2%)	76 (41.8%)	132 (54.8%)	0.011
– Lectures in school	176 (41.6%)	66 (36.3%)	110 (45.6%)	0.059
– Health talk in church/mosque	41 (9.7%)	15 (8.2%)	26 (10.8%)	0.411
– Social media (Facebook, WhatsApp, Twitter, Instagram)	25 (5.9%)	12 (6.6%)	13 (5.4%)	0.679
– Others (Women’ meeting = 5, In a travelling bus = 3)	8 (1.9%)	6 (3.3%)	2 (0.8%)	0.080
**What drugs are used for periodic deworming in preschool children?** ^**a**^ ***N*** **= 423**				
– Albendazole (Zolat®, Zeben®, Avis®, Wormplan®)	246 (58.2%)	117 (64.3%)	129 (53.5%)	0.029
– Mebendazole (Wormin 100®)	216 (51.1%)	102 (56.0%)	114 (47.3%)	0.078
– Pyrantel (Combantrin®, Ascatrin®, Combiworm®)	244 (57.7%)	87 (47.8%)	157 (65.2%)	0.001
– Others (Flagyl = 5, Antibiotics = 2, Blood tonics = 6)	13 (3.1%)	7 (3.8%)	6 (2.4%)	0.571
– I have no idea (Doctor, Nurse or PPMV gives me drugs)	111 (26.2%)	50 (27.5%)	61 (25.3%)	0656
**How often should preschool children be dewormed?** ***N*** **= 423**				
– Every 2–3 months ^1^	44 (10.4%)	24 (13.2%)	20 (8.3%)	0.000 ^1,2^
– Every 4–6 months ^1^	328 (77.5%)	150 (82.4%)	178 (73.9%)	
– Every 1–2 years ^2^	40 (9.7%)	7 (3.8%)	33 (13.7%)	
– Only when my child has symptoms ^2^	2 (0.5%)	0 (0.0%)	2 (0.8%)	
– I have no idea ^2^	9 (2.1%)	1 (0.6%)	8 (3.3%)	
**Where did you obtain the last deworming treatment?** ***N*** **= 363** ^**b**^				
– Public Hospital/PHC	219 (60.3%)	98 (53.8%)	121 (66.9%)	0.002
– Private hospital/clinic	35 (9.6%)	20 (11.0%)	15 (8.3%)	
– Patent and Proprietary Medicine vendors (Chemist store)	103 (28.4%)	64 (35.2%)	39 (21.5%)	
– Others (Church-based deworming campaign, *n* = 6)	6 (1.7%)	0 (0.0%)	6 (3.3%)	
– I have never dewormed my preschool child ^b^	70 ^b^			

^a^More than one answer may apply – as mothers were asked to affirm all the options that apply to them

^b^Mothers who have never dewormed their preschool children (*n* = 70) were excluded from this Chi-Square statistics

^1^These responses: every 1–2 months and every 3–6 months, were aggregated for Chi-Square statistics

^2^These responses: every 1–2 years, only when my child has symptoms, & I have no idea, were aggregated for Chi-Square Statistics

There was also significant difference in knowledge of the frequency of periodic deworming (*p* = 0.029) between mothers who periodically dewormed their PSAC and mothers who did not. Furthermore, there was a statistically significant difference in the facilities mothers obtained their last treatment for preventive chemotherapy between mothers who periodically deworm their PSAC and those who did not (*p* = 0.002).

Although mothers in the FGD sessions knew they can always source anthelmintics from the primary health center/hospital or from the proprietary and patent medicine vendors (PPMV), there were some misconceptions on the correct frequency of deworming. A few mothers (two mothers) contended that since they only allowed their PSAC limited access to sugary and sweet foods, they could not be infected and should not be taking PC; while three mothers argued that their PSAC will get the treatment for free when they (PSAC) start school.

*“… My last child is 4 years, she got the worm drugs last year when some doctors shared drugs in our church, but she will start school this September, and she will get the drugs like her brothers in school…”.* Mrs. NO, FGD

### Mothers’ attitude to periodic deworming in PSAC

**Fig. 1:**
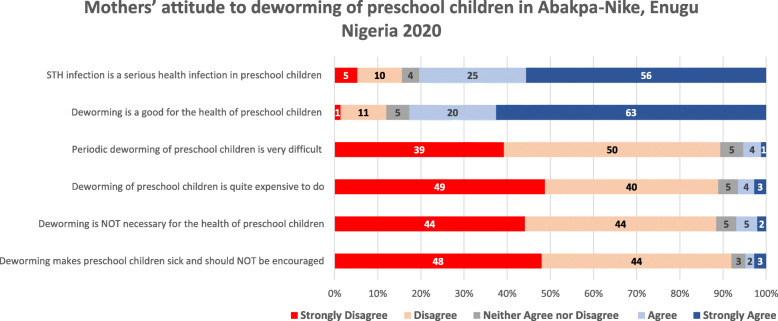
Mothers’ attitude to periodic deworming of preschool children in Abakpa-Nike, Enugu Nigeria 2020

Most mothers (81%) considered STH infections a serious health issue in PSAC and 83% considered periodic deworming is good for the health of PSAC. About 89% of mothers disagreed that periodic deworming of PSAC is both difficult and expensive. Other attitudinal views of mothers to periodic deworming of PSAC are shown in Fig. 1. There was a general positive attitude to deworming of PSACs to cure STH infection. However, two mothers expressed reservation on the safety of repeatedly giving the same drugs to children, four mothers questioned lack of available STH vaccines like for other childhood illnesses, and 11 mothers questioned (after health education talks at the end of each FGD session) why mothers were not given PC like SAC.

*“… I do not understand. for how long I should be giving them (preschool children) these drugs? my children do not like wearing shoes and they play outside all the time. Is this drug safe?”* Mrs. BO, FGD

*“… to be continuously giving children medicine/drug for worm is tiring, why can’t the hospital give immunization for it like the immunization for measles we come here to get …”* Mrs. IN, FGD

*“… Doctor, from what you explained, these drugs are good for everybody to take every three months, why is the government not giving all of us as they give the children in primary school?”* Mrs. IA, FGD

Two in five (40.0%) mothers who had never dewormed their PSAC (*n* = 70) saw no need to deworm their children as they children will get dewormed when they start school, while about 27% of these mothers explained that their children did not need deworming as they do not take excessive sugar or sugary food/drinks – Supplement Table 1. About two in three households (67.4%) treated their drinking water and about half (50.6%) of the PSACs seldomly wore footwear during the day – Supplement Table 2.

Bivariate analysis of mothers’ attitude to periodic deworming with their periodic deworming practices showed that mothers who did not consider STH infections in PSAC as a serious infection, nor considered deworming good for PSAC were less likely to periodically deworm their PSAC; OR = 0.57 (*p* = 0.035) and OR = 0.48 (*p* = 0.007), respectively. Results of other bivariate analyses are shown in Table 4. Results of multivariable logistics regression analysis – Table 5, showed that mothers who did not periodically deworm their PSAC were less knowledgeable on the modes of transmission of STH infections (AOR = 0.62, 95% CI: 0.48–0.81, *p* = 0.000); complications of STH infections (AOR = 0.77, 95% CI: 0.61–0.98, *p* = 0.034); and accurate frequency for periodic deworming (AOR = 0.41, 95% CI: 0.18–0.90, *p* = 0.026).

**Table 4:** Association between mothers’ attitude to STH infections and deworming to periodic deworming practice in preschool children in Abakpa-Nike, Enugu, Nigeria, *N* = 433

To what extent do you agree or disagree with these statements	Total (%) (***N*** = 433)	Periodic deworming of Index child		Crude OR (95% CI)	***P***-value
		Yes (***n*** = 182)	No (***n*** = 251)		
**STH infection is a serious infection in preschool children**					
– Agree + Strongly Agree	350 (80.8%)	156 (85.7%)	194 (77.3%)	0.57 (0.34–0.94)	0.035
– Indifferent + Disagree + Strongly Disagree	83 (19.2%)	26 (14.3%)	57 (22.7%)		
**Deworming for STH is good for preschool children**					
– Agree + Strongly Agree	358 (82.7%)	161 (88.5%)	197 (78.5%)	0.48 (0.28–0.82)	0.007
– Indifferent + Disagree + Strongly Disagree	75 (17.3%)	21 (11.5%)	54 (21.5%)		
**Periodic deworming of preschool children for STH infections is very difficult to practice**					
– Agree + Strongly Agree	23 (5.3%)	8 (4.4%)	15 (6.0%)	1.38 (0.57–3.33)	0.522
– Indifferent + Disagree + Strongly Disagree	410 (94.7%)	174 (95.6%)	236 (94.0%)		
**Periodic deworming of preschool children for STH infections is very expensive to practice**					
– Agree + Strongly Agree	28 (6.5%)	10 (5.5%)	18 (7.2%)	1.33 (0.60–2.95)	0.556
– Indifferent + Disagree + Strongly Disagree	405 (93.5%)	172 (94.5%)	233 (92.8%)		
**Periodic deworming for STH infections is not necessary for preschool children**					
– Agree + Strongly Agree	30 (6.9%)	5 (2.7%)	25 (10.0%)	3.92 (1.47–10.44)	0.004
– Indifferent + Disagree + Strongly Disagree	403 (93.1%)	177 (97.3%)	226 (90.0%)		
**Deworming for STH infections makes preschool children sick**					
– Agree + Strongly Agree	17 (3.9%)	5 (2.7%)	12 (4.8%)	1.77 (0.62–5.14)	0.326
– Indifferent + Disagree + Strongly Disagree	416 (96.1%)	177 (97.3%)	239 (95.2%)		

*Abbreviations*: *OR* Odds ratio, *CI* Confidence interval

**Table 5:** Multivariable logistics regression analysis of mothers’ familiarity with and specific knowledge of STH infections and frequency of periodic deworming of preschool children in Abakpa-Nike, Enugu, Nigeria, *N* = 433

Knowledge on different STH, transmission, symptoms, and complications of STH infection in preschool children	Mothers who periodically deworm (***n*** = 182)	Mothers who do NOT periodically deworm index child (***n*** = 251)		***P***-value
		OR	95% CI	
**Are you familiar with soil-transmitted helminthiasis?**				
– Adjusted OR ^a^	Reference	0.00	0.00–0.00	0.999
**Knowledge of the different STH infections in preschool children.**				
– Adjusted OR ^a^	Reference	0.94	0.67–1.32	0.738
**Knowledge of the modes of transmission of STH infection in preschool children**				
– Adjusted OR ^a^	Reference	0.62	0.48–0.81	0.000
**Knowledge of the different symptoms of STH infection in preschool children**				
– Adjusted OR ^a^	Reference	0.76	0.57–1.02	0.069
**Knowledge of the main complications of STH infections in preschool children.**				
– Adjusted OR ^a^	Reference	0.77	0.61–0.98	0.034
**How often should a preschool child be dewormed for STH infections?**				
– Adjusted OR ^a^	Reference	0.41	0.18–0.90	0.026
**Do you know Albendazole is used for periodic deworming of preschool children**				
– Adjusted OR ^a^	Reference	1.00	0.46–2.11	0.972
**Do you know Mebendazole is used for periodic deworming of preschool children**				
– Adjusted OR ^a^	Reference	0.28	0.09–0.90	0.031
**Do you know Pyrantel is used for periodic deworming of preschool children**				
– Adjusted OR ^a^	Reference	8.03	2.22–29.03	0.001

*Abbreviations*: *OR* Odds ratio, *CI* Confidence interval

^a^ ORs were adjusted for sociodemographic factors: mothers age, marital status, mothers educational status, mothers occupation, fathers occupation, religion, family monthly income, number of children < 5 years in the family, sex of the index child, source of information on STH and deworming

## Discussion

Overall coverage of periodic deworming in PSAC in this setting is low – about 42% (95% CI: 37.3–46.8%), which is comparable to 44.8% reported in Alakahia community, Rivers State, Nigeria [31]. This is not a surprise given that current strategies for mass deworming PSAC are not as widely known as the school-based deworming programs which specifically targets SAC [8, 16]. Current strategies for PSAC rely heavily on health facilities-based child health programs and occasional programs for other neglected tropical diseases [12]. Given that PSAC are a key population for STH burden, potential for long-term health and educational consequences and their contribution to ongoing transmission, our study adds to the growing evidence that relying on this existing strategy for STH control in PSAC runs the risk of missing WHO STH elimination target of 2030 [12, 18].

Our study shows that specific knowledge on the transmission, symptoms, and complications of STH infections are significantly associated with PC practice of PSAC – which is similar to findings in Ile-Ife, Nigeria [32], Ibadan, Nigeria [33], Jimma, Ethiopia [34] and KwaZulu-Natal, South Africa [35]. Although the overwhelming majority of mothers in our study are familiar with STH, specific knowledge on the transmission, symptoms and complications is deficient. Most mothers in our study were first familiar with STH during visits to the hospitals for STH related illness or some other illness. During such visits, mothers are succinctly informed about worms and often given treatment. Brief health education interactions such as these do not adequately address gaps in mothers’ specific knowledge of the different STH, transmission, symptoms, and complications of STH infections. Unfortunately, such brief interactions during visits to health facilities are the only health education opportunities many mothers in this setting have.

Misconceptions on the epidemiology of STH infections are rife among mothers in our study setting have serious health consequences. Widespread misbeliefs that consuming sugary food including banana, pineapple, orange, and soursop causes or exacerbates STH symptoms not only hamper health-seeking behaviours for STH infections but could potentially lead to vitamin deficiency in PSAC. For instance, some mothers strongly believed that since they do not feed their children with such sugary food and sweet fruits, there is no need to deworm their preschool age children. A similar finding was also reported among caregivers in Alakahia community, Rivers State Nigeria [31]. Another misconception that children are born with STH infections or acquire it from breastmilk prevents mothers from taking adequate protective measures to avoid STH infections in their PSAC. These misconceptions and beliefs further undergird the need for comprehensive health education interventions as integral components of STH control in these settings [35].

This study also reveals differences in the relationship between mothers’ knowledge of specific anthelmintics and deworming frequency in PSAC. Albendazole and Pyrantel are the two most commonly known anthelmintics among mothers in our study. However, while albendazole is more commonly known by mothers who periodically deworm their PSAC, Pyrantel is more known by mothers who do not deworm their PSAC. Pyrantel, widely marketed as Combantrin® (Pyrantel Pamoate) has been longer on the Nigeria market [31], and is widely marketed on national televisions and radios. Indeed, Combantrin is well-known even among mothers who had never dewormed their children in our FGD sessions. However, as our study shows, a strong brand name does not necessarily translate to adherence to periodic deworming. Although we did not find any economic explanation for this disconnect in our FGD sessions, Stanley et al. suggests that economic impediments could contribute to this [31].

This study also shows that mothers who periodically deworm their children report a more accurate frequency of deworming than mothers who do not periodically deworm their PSAC. It follows that as mothers are more knowledgeable of the transmission of STH infections, they better appreciate the ever-present risk of their children getting infected with STH [16, 35]. Also, as these mothers are more knowledgeable on the symptoms of STH infections, they are more likely to suspect STH infections in their children and deworm accordingly, and even incur out-of-pocket expenses at private hospitals and patent medicine vendors (Chemist stores) to do so.

Fortunately, our study shows that there is a general positive attitude towards deworming among mothers in our study. Our survey data and FGD sessions reveal that poor adherence to periodic deworming is squarely due to poor specific knowledge of the epidemiology and pathology of STH infections and not due to negative attitudinal perspectives per se. In our FGD sessions, we observed that stark ignorance of the disease in the community has led to some misconceptions that has cascaded across generations. These misconceptions were effectively dispelled with the health education sessions we had at the end of the FGD sessions. Health education/promotion interventions must be developed to match divergent health literacy levels of mothers in this setting.

Despite rigorous efforts to help mothers recall the deworming history of their children in the community survey, some elements of recall bias could still impact our study results. Although we used a qualitative approach to collect nuanced views and opinions of mothers in the study, our sample size for the FGDs was small. Furthermore, the study was conducted in an urban slum, though representative of its population, the study findings might not represent more affluent communities.

The WHO estimates (in 2018) that over 270 million PSAC and school-aged children (SAC) in sub-Saharan Africa countries endemic to STH require periodic chemotherapy (periodic deworming) for STH infections [15]. Out of these, over 20 million PSAC in Nigeria (2018 estimates) require periodic chemotherapy [15]. Program coverage for this age group in Nigeria, as in many other low- and middle-income countries, have failed to reach the 75% target set by WHO [15, 19, 36]. These shortages are unlikely to be corrected without significant changes in the current strategies to reach this high-risk age group [19]. Our study findings demonstrates that in addition to the current interventions on water, sanitation and hygiene, interventions to improve mothers’ and caregivers’ awareness of and attitude to periodic deworming of their PSAC in these highly endemic settings could be helpful in achieving the World Health Organization’s goal of eliminating STH infections in PSAC and SAC by 2030 [11].

## Conclusions

Significant gaps in mothers’ and caregivers’ perception of periodic deworming is a major barrier to periodic self-deworming of PSAC in this setting. Periodic self-deworming of PSAC by their mothers and caregivers will continue to be hampered by mothers’ poor perception of the burden and transmission of the disease. Health education on the burden and transmission of STH infections could complement existing health strategies to improve periodic deworming coverage of PSAC in this setting.

## Supplementary Information

**Additional file 1: Table S1**. Reasons mothers gave for having never dewormed their preschool children in Abakpa-Nike, Enugu Nigeria 2020. **Table S2.** Mothers preventative practices against STH in preschool children in Abakpa-Nike, Enugu Nigeria.

## Data Availability

The dataset generated and analyzed in this study is freely available from the corresponding author on reasonable request.

## Abbreviations

FGD: Focal group discussions
LMIC: Low- and middle-income countries
PSAC: Preschool age children
SAC: School age children
STH: Soil-transmitted helminths
WASH: Water, Sanitation and Hygiene
WHO: World Health Organization
