# Supplementary material for: Perception and attitudinal factors contributing to periodic deworming of preschool children in an urban slum, Nigeria
Source: BMC Public Health. 2020 Dec 1;20:1839. doi: 10.1186/s12889-020-09958-x (PMC7708184; doi:10.1186/s12889-020-09958-x)
Supplement: Supplementary file 1 — Additional file 1: Table S1. Reasons mothers gave for having never dewormed their preschool children in Abakpa-Nike, Enugu Nigeria 2020. Table S2. Mothers preventative practices against STH in preschool children in Abakpa-Nike, Enugu Nigeria. [file 12889_2020_9958_MOESM1_ESM.docx]

**Supplement Table 1: Reasons mothers gave for having never dewormed their preschool children in Abakpa-Nike, Enugu Nigeria 2020**

| **Reasons mothers gave for having never dewormed their preschool children** | **N = 70** | **Percentage** |
| --- | --- | --- |
| **If you have never dewormed your child, why?** |  |  |
| - Deworming is harmful | 4 | 5.7% |
| - Deworming is too expensive | 5 | 7.1% |
| - My child will be dewormed when he/she starts school | 28 | 40.0% |
| - My religion forbids it | 1 | 1.4% |
| - Not necessary because my child does not take too much sugar | 19 | 27.1% |
| - I never knew I am supposed to deworm my child | 9 | 12.9% |
| - Claimed to have dewormed their child using herbal preparations | 4 | 5.7% |

**Supplement Table 2: Mothers preventative practices against STH in preschool children in Abakpa-Nike, Enugu Nigeria**

| **Preventative measures practiced at home** | **Frequency**  **N = 433** | **Periodic deworming of Index child** | | **P-value** |
| --- | --- | --- | --- | --- |
|  |  | **Yes**  **(n = 182)** | **No**  **(n = 251)** |  |
| **Which of these STH preventive measures do you practice at home?** |  |  |  |  |
| - Wash hands after each toilet use | 393 (90.8%) | 164 (90.1%) | 229 (91.2%) | 0.738 |
| - Wash hands before each meal | 370 (85.5%) | 151 (83.0%) | 219 (87.3%) | 0.217 |
| - Wash vegetables and fruits before eating | 365 (84.3%) | 149 (81.9%) | 216 (86.1%) | 0.284 |
| - Treating drinking water | 293 (67.4%) | 117 (64.3%) | 175 (69.7%) | 0.254 |
| - Thoroughly cooking meat before eating | 347 (80.1%) | 139 (76.4%) | 208 (82.9%) | 0.113 |
|  |  |  |  |  |
| **Who does toilet care for your child** |  |  |  |  |
| - Myself | 316 (73.0%) | 140 (76.9%) | 176 (70.1%) | 0.018 |
| - The child | 99 (22.9%) | 31 (17.0%) | 68 (27.1%) |  |
| - Others in the family (elder sibling, father, aunt/uncle) | 18 (4.2%) | 11 (6.0%) | 7 (2.8%) |  |
|  |  |  |  |  |
| **How often do you trim you children’ fingers** |  |  |  |  |
| - Every 1 week | 272 (62.8%) | 101 (55.5%) | 171 (68.1%) | 0.064 |
| - Every 2 weeks | 57 (13.2%) | 28 (15.4%) | 29 (11.6%) |  |
| - Every 1 month | 6 (1.4%) | 3 (1.6%) | 3 (1.2%) |  |
| - Whenever it grows | 98 (22.6%) | 50 (27.5%) | 48 (19.1%) |  |
|  |  |  |  |  |
| **How often does my child wear shoes during the day?** |  |  |  |  |
| - All the time | 79 (18.2%) | 35 (19.2%) | 44 (17.5%) | 0.127 |
| - Most times | 135 (31.2%) | 65 (35.7%) | 70 (27.9%) |  |
| - Not Often | 219 (50.6%) | 82 (45.1%) | 137 (54.6%) |  |
